# An examination of knowledge, attitudes and practices related to lead exposure in South Western Nigeria

**DOI:** 10.1186/1471-2458-6-82

**Published:** 2006-03-29

**Authors:** Eugenious O Adebamowo, Oluwole A Agbede, Mynepalli KC Sridhar, Clement A Adebamowo

**Affiliations:** 1 Department of Civil Engineering, University of Ibadan, Ibadan, Oyo State, Nigeria; 2 Department of Epidemiology, Medical Statistics and Environmental Health, College of Medicine, University of Ibadan, Ibadan, Oyo State, Nigeria; 3 Department of Surgery, College of Medicine, University of Ibadan, Ibadan, Oyo State, Nigeria

## Abstract

**Background:**

Lead is a highly toxic and pervasive metal. Chronic exposure to low levels is responsible for significant health effects, particularly in children. Prevention remains the best option for reducing childhood lead exposure, however the knowledge, attitudes and practices to lead exposure in many developing countries is not known.

Methods: We conducted four focus group discussions (FGD) to evaluate knowledge attitudes and practices to lead exposure in Nigeria. An FGD guide was developed from the literature and preliminary discussion with members of the public. Participants in the FGD were randomly selected from adults living in Ibadan, South Western Nigeria in 2004.

**Results:**

We found that there was limited awareness of the sources of lead exposure in the domestic environment and participants had little knowledge of the health effects of chronic low-dose lead exposure.

**Conclusion:**

We conclude that the findings of this study should be used, in conjunction with others, to develop appropriate health education intervention for lead exposure in the domestic environment.

## Background

Lead is a ubiquitous and poisonous heavy metal. It is widely distributed in the environment (air, soil, sediment, surface and ground water, food, dust, paint) and in biological systems [1]. It occurs both naturally and as a result of human activities [2]. One of the most common sources of human exposure to lead is through exhaust from use of leaded petrol. However this practice is being discontinued worldwide [3] leaving the domestic environment (through house paint, potable water and dust) as the main continuing source of lead exposure in many communities around the world. While both adults and children can suffer from chronic low-dose lead exposure, the effect is more marked in children [2,4-6].

In Nigeria, like most developing countries, very little attention is currently paid to environmental health problems including chronic lead exposure [7]. Yet these factors are responsible for more morbidity, disability-adjusted quality of life loss and mortality than in developed countries [7-9]. It was recently estimated that a reduction of the blood lead levels of children in the United States from 17.1 μg/dL to 2.0 μg/dL, in the period 1976 to 1999, resulted in public health benefits of $319 billion [10]. The proportional impact of reducing childhood lead exposure in developing countries, where children's blood lead levels are likely to be higher, would be much greater. While many developing countries are currently making efforts to reduce exposure to lead by using lead-free petrol, very little is being done to address the more ubiquitous sources of exposure in the domestic environment [9]. In this study, we conducted several focus group discussions with adult residents of Ibadan, a large metropolis in South Western Nigeria to evaluate their knowledge, attitudes and practices with respect to chronic low-dose domestic lead exposure and its effect on health.

## Methods

This study was conducted in Ibadan, South Western Nigeria in 2004, as part of the Reducing Exposure of Children to Lead Study (RECLES) of the University of Ibadan. Nigeria has a population of about 136 million people, at least 40 million of whom live in the South Western part of the country. Most, 60%, of the population live below poverty line and the estimated GDP per capita in 2004 was $1,000. Ibadan is one of the major urban centers in Nigeria; the largest indigenous city in West Africa with a population of about 2 million people. The population is largely engaged in small scale farming, trading and service occupations. Ibadan hosts several institutions of higher learning, of which the University of Ibadan, established in 1948, is the oldest. Along with its affiliated medical institution, the University College Hospital; the two institutions formed the base for this study. About 80% of the adult population of Ibadan is literate. There are several small and medium scale industries in Ibadan and during the 1990s, there was a car battery manufacturing company, but it closed down about 8 years ago.

In order to ensure representation of the population of the entire city, we randomly selected 40 healthy individuals by personal contact and mail, from the 5 administrative units of the city, taking care to ensure balance of religious affiliation, occupation, gender and social economic status. The individuals were invited to meet for a discussion on a pertinent health problem and were not told before hand that the topic of the discussion would be lead exposure. Recruitment continued until there was a group of ten people for each of the four groups. After arrival, participants were informed about study objective and told that each interview will last about 11/2 hours. Trained facilitators conducted the sessions and written consent was obtained from all focus groups participants. Individuals younger than 18 years of age, those unable to communicate in English or Yoruba (the indigenous language of Ibadan) or those unable to give consent were excluded from the study. Participants were given gifts worth $7:00 to cover transport and other expenses incurred in order to participate in the study.

The discussion guide for the focus group discussions was developed by EOA and CAA based on the literature and knowledge of the local environment. Facilitators occasionally interjected in the discussions using a non-directive approach to focus participants on the topic of interest and move discussions along. Following introduction, participants were encouraged to freely discuss along the themes in the discussion guide shown in Table 1. The discussions were audio-taped and transcribed by a secretary who was not part of the study team. The transcripts were then reviewed by the authors in a two step process. First, major themes were identified and these were aggregated into lists. Phrases and quotations that highlighted these themes were identified. Sub-themes within the major themes were also identified and aggregated into lists. The major themes and sub-themes from each reviewer were then compared and the lists merged. Where there were disagreements between the raters, a third person was asked to review the pertinent sections of the transcript and a consensus reached on the substance. Coding began by identifying broad conceptual themes like; knowledge of lead and lead exposure, attitudes to lead exposure, health implications and practices regarding lead exposure. Specific attention was given to the knowledge about lead, lead level testing in the home, lead poisoning and the health impact of lead exposure. Ethical approval for this study was obtained from the Oyo State Ministry of Health Ethics Committee.

## Result

There were 39 participants overall, with one person failing to keep his appointment at the venue of the focus group discussions. The age of the participants ranged from 23 to 72 years with mean (SD) of 44.5 (12.8) years. There were 19 (49%) women and 20 (51%) men; 6 Muslims (15%) and 33 Christians (85%) and their occupations were students (9, 23%), teachers (6, 15%), drivers (6, 15%) and clerks (6, 15%). The rest belonged to a wide variety of occupations.

### Knowledge

#### What is lead and where can it be found?

Most participants believed that lead is a metal which is used in soldering materials and it can be found in cooking pots, domestic and automobile batteries, pencils, cosmetics, fuel, local herbs and medications. Discussants thought that lead is poisonous. It can cause asthma, cancer, eye and chest diseases and can shorten lifespan. Many participants mentioned some of the commonly used canned foods and condiments in Nigerian markets, such as canned tomato puree, canned fish, tinned milk, cutlery and cooking utensils as potential sources of lead.

*Participant: My own understanding of lead is both abstract and physical. The physical is in form of gold, silvery in color, and usually heavy in substance. It is used for soldering flexible wires, radio and television (...) used by blacksmith, tinkers, and other likes *(sic).* The other one which is abstract so to speak is within the mineral sector and my own overall understanding of the thing is that it is poisonous because it is usually incubated through liquid water or sucking lead pencil or eating any kind of tinned food that has expired thereby secreting lead into the container which is made of tin as a component *(sic).* There is another one that can be absorbed through water, depending on the kind of soil the water flows through *(sic).

*Participant: In all this small small batteries we use for torch light, there is lead in it, it is like a rod (inside) the battery*(sic)...*People also use it *...*like those selling herbs (alternative medicine practitioners). If you have itches, scratches or any infection in the body, you go to buy soap for it; they will mix it with (the) soap and sell it to you. I even used it for my own grand daughter when she had rashes on her head*.

*Participant: *...*lead is in this pencil that we use to write*.

*Participant: It is a heavy metal and we can find it in traces of fumes of vehicle *(sic).* It affects the health of children, that is why if you see some children in the park ... they don't behave like normal children ... when they inhale it in large quantity it can affect health *(sic).

#### What risks are associated with exposure to lead?

Most of the discussants stated that they believe that lead is poisonous and dangerous to health but there was no unanimity about which of the body's systems is vulnerable. Many of the participants thought exposure to lead can cause cancer.

*Participant: It can affect the eyes, that is why they (workers who use lead products) cover the eyes with face mask (goggles) *(sic).

Facilitator: Many of you mentioned pencil as a source of lead, has anyone noticed anything peculiar about children using pencil?

*Participants: I see children playing with pencil. I have never paid attention and I have never met a mother in my life (who) complained about pencil apart from the sharpness *(sic).* When I was a child I used to play with pencil and put (it) in my mouth, nothing has ever happened to me *(sic).

*Participant: Yes, I have 2 experiences, one was with a child sucking lead pencil and it resulted in upset stomach *(sic).* She was hale and hearty before sucking the substance. The other one also resulted in stomach upset by playing with battery and licking the head, thereby absorbing the water from the battery *(sic).* So in the two instances, it ultimately led to stomach pain*.

*Participant: It can shorten life because it can affect any organ. We know it is bad, we don't know the exact thing it can cause in the body *(sic).

*Participant: I noticed this welder (who) uses lead ... it makes them lean *(sic).* It affects their health*.

*Participant: (Children) tend to be dull if they are exposed to lead*.

*Participant: It affects intelligence*.

Where can we get information about lead and additional comments?

Discussants felt that they can obtain information about lead exposure from welders, automobile battery repairers and manufacturers. There was general agreement on the need for the government to do more to increase awareness of lead exposure while putting effective remediation measures in place (Table 2).

### Attitudes

#### What do you think of when you hear "lead poisoning"?

The use of the term poison engaged the attention of the discussants, though they did not know specifically about lead poisoning. They were sure that the metal is poisonous. Overall, they felt that apart from specialists in the field, most people do not know what "lead poisoning" is about.

#### How do you think we are exposed to lead and how can we reduce it?

Most of the discussants believed that one can be exposed to lead through petrol, pipe-borne water and pencils. Less commonly, they also mentioned soil, cooking utensils, soap and industrial pollution as sources of lead exposure. There was general agreement that there is a need to increase awareness of lead exposure and increase the role of regulatory agencies in ensuring that products sold on the market do not contain lead. Some participants suggested that people should return to the use of earthenware pots for cooking, out of concern that modern cooking utensils may be associated with lead exposure, though they acknowledged that such a move is not likely to be popular. Other participants thought that recent government efforts to reduce importation of old vehicles (used vehicles are commonly imported into Nigeria and constitute the majority of new vehicle purchases) will reduce the amount of lead pollution from exhaust of old cars with inefficient engines.

### Practices

#### Have you tested the amount of lead in your environment? Would you accept to have your environment tested for lead?

None of the participants has ever tested their environment for lead. A participant, who works in the state government's department in charge of housing and civil engineering works, was aware that the department has the capacity to test lead level in soil and does it for corporate bodies and large organizations before they are given building permits.

*Participant: supposing what you are going to use to test the level is going to have a negative impact. (Secondly) what use will the test be to (an individual). ...a man whose daily income depends on constant exposure to lead will have been exposed for too long and (he will say) I am now used to it. ... it needs ... education, letting them know why, is there going to be harm *(sic).

*Participant: Nowadays you can never know the purpose of research because we have read in some papers that some people will just bring something in their environment because they want to make money or name out of it *(sic).

*Participant: I think the first and best solution is awareness. I think the first solution is awareness for people to know that they have a lot of lead in their environment and they have to reduce it *(sic).

*Participant: The reason I am opposing awareness is that ... we have a lot of poverty *(sic)*. If you are saying there should be awareness, are you saying that the pot (we use for cooking) we have should be thrown away (if it is found to contain lead)?*

*Participant: I think awareness should be done. Like in the primary and secondary school level, this kind of study should be included in the school curriculum so that the generation coming will be able to have proper knowledge of what is going on in their environment *(sic).

*Participant: paints in homes, because we are even advised that when you paint a house, if possible, let people not be there for a few days *(sic).

*Participant: ... thorough research (we) will be able to bring out certain results which I believe will be passed to the government and invariably ... legislation will result. There are some soap(s) that contain heavy metal, if we know such ones so as to avoid them *(sic).

Some participants think that since lead is used for welding, pencils, paints, etc, it is needed and beneficial. (Table 3)

## Discussion

In this study, we found some awareness of lead exposure among our participants. Many of them were aware of the presence of lead in petrol but had little knowledge of domestic sources of lead exposure such as paint, water and soil. Many of the participants confused the popular appellation for pencils, which is "lead pencil", to imply that the writing element in pencils were made of lead. Several of our FGD participants were aware of lead exposure arising from car battery manufacture and repairs. There used to be a motor car battery manufacturing plant in Ibadan and many residents were aware of the environmental degradation associated with its operations. Participants were also aware of the presence of lead in some alternative medicines and in association with occupations such as welding. Some of our participants were aware of the health implications of lead exposure in children, suggesting that it may be responsible for "abnormal behavior" and "dullness". Nevertheless, none of the participants has ever tested their environments for lead.

At least one participant who works with the government department in charge of civil engineering was aware that facilities for testing lead level in the environment exists but added that this was usually done by large organizations and not individuals. Participants believed that there is need to increase awareness of lead exposure in the community. Many however agreed when another participant suggested that people are likely to be pragmatic in their response to any campaign to reduce exposure to lead, suggesting that alternative sources of income should be found for those whose occupation is likely to be affected by lead remediation activities, otherwise such campaigns will fail. Furthermore, it was suggested that alternatives should be provided for lead contaminated products. While some of the participants were hopeful that research results will lead to government intervention, others were not as optimistic, suggesting that previous experience does not support any expectation that the government will respond positively to such research. Most of the participants felt that they can obtain information about lead from those who are occupationally exposed to it.

There is increasing awareness of the risks posed by domestic exposure to lead, particularly to children. Children can be exposed to lead through dust inhalation and ingestion [10,11]. In a survey of households in the United Kingdom, the total estimated lead intake of young children was 36 μg/day, of which 1 μg/day was by inhalation and the rest by ingestion [12]. Recent prevalence studies show that over 90% of children in urban and rural communities of Cape Province, South Africa have blood lead levels ≥ 10 mg/dl. Studies in other countries likewise suggest that childhood lead poisoning is a widespread urban health problem throughout the continent of Africa [13,14]. Reduction of childhood lead exposure will result in substantial economic gains, possibly to a greater degree than has been reported from developed countries [10,15].

This study has outlined the current knowledge, attitudes and practices of a cross section of Ibadan residents about lead exposure. To our knowledge, there is no previous report on the use of FGD to ascertain knowledge of health hazard posed by lead exposure in Nigeria or any other parts of Africa. Our participants were similar to the general Nigerian population in terms of age, sex and occupation [16]; however the presence of a battery manufacturing company in Ibadan in the recent past may have increased the baseline knowledge of residents in this city to lead exposure compared to other parts of Nigeria. There is little or no enforcement of minimum standard for lead content of domestic environment in Nigeria. This is partly because of low awareness of the health implications of these exposures and competing attention from infectious diseases like HIV/AIDS and malaria. There has never been domestic lead abatement in Nigeria and none is planned.

Focus group discussions provide an opportunity to interview a group of individuals in a directed conversation about a specific topic and it can be used to generate new insights about attitudes and beliefs [17,18]. The interaction among participants leads to the promotion of rich discussion and opportunity to present contrary opinions that are not limited by the constraints imposed by the limited choices in a quantitative study [17,19]. In situations where little previous documentation exists, such as this topic, focus group discussions help to generate new ideas and hypothesis for further research. They can also be used in conjunction with other methodological techniques for triangulation purposes thus helping to validate research findings [20]. However like other qualitative research methods, their results and conclusions must be treated with caution [21].

We conducted four focus group discussions and it may be considered that bigger groups or more groups would lead to more valid conclusions. This is however not necessarily so [22,23]. In addition, it is possible that having such a heterogeneous group may dilute the information obtained and may be unrepresentative of the population's knowledge and attitudes to lead exposure. The facilitators' prompts and interventions may also be misunderstood and in the few instances where the participants spoke in Yoruba, the sense of the contribution may have been lost in the translation.

## Conclusion

In conclusion, this study shows that there was limited knowledge and awareness of domestic sources of lead exposure and its health effect in Nigeria. The origins of popular misconceptions about lead exposure and effective means of correcting them need to be explored. The most popular of these was about pencils, a ubiquitous writing implement made of graphite that carries no known health risk. Our findings suggest that more studies are needed to fully understand the knowledge, attitudes and practices of this population to lead exposure in order to develop appropriate health education intervention.

## Competing interests

The author(s) declare that they have no competing interests.

## Authors' contributions

EOA conceived the study and the design, collected the data, analyzed the data and contributed to drafting the manuscript

OAA participated in the design of the study and contributed to drafting the manuscript

MKCS participated in the design of the study and contributed to drafting the manuscript

CAA participated in the conception and design of the study, analyzed the data, contributed to drafting the manuscript and provided funding for the study

## Pre-publication history

The pre-publication history for this paper can be accessed here:



## Figures and Tables

**Table 1:** Excepts from the Focus Group Discussion Guide used for the study of Health Effects of Lead Exposure, Ibadan, 2004

**Number**	**Questions**	**Probes**
1.	What comes to mind when you hear the word "lead"?	What do you think it is? Where do you think it can be found? What risks do you think is associated with exposure to lead?
2.	Have you ever tested the lead level in your domestic environment? [IF "YES"] Why did you do the test?	What are the reasons you might participate to do a lead level test [or not]
3.	What images does the word "lead poisoning" bring up for you or what do you think of when you hear the word "lead poisoning"?	What about lead exposure? What other words have similar meanings for you?
4.	What impact do you feel lead or lead poisoning have on health? What (other) factors do you believe contribute to Health? What do you believe about lead exposure and how to Reduce it? Concerns? Perceived risks? Hopes? Perceived benefits?	
5.	What kinds of things would be important to you in deciding what to do about lead exposure?	What are the reasons you might participate [or be concerned about lead]? What are the reasons you might not be concerned about lead? Where do you think you can find information about lead exposure in your community?
6.	What kinds of methods of reducing lead exposure do you know?	

**Table 2:** Participants' knowledge about lead exposure in Ibadan, Nigeria 2004

Domain	Responses	
	
	Common Responses	Less Common Responses
What comes to your mind when you hear the word lead? What do you think it is?	• Lead is a metal • Lead is used for soldering • Lead is a mineral resource	• Lead is used to make cooking pots • Lead is used to write • It is mixed with local soap for bathing • Lead is used to make batteries • Lead is poisonous
Where can lead be found? What do you think it is?	• It can be found in battery(automobile and domestic)	• Lead is pencil • Lead is a heavy metal • It can be found in cosmetics • It can be found in soil • It can be found in pencils • It can be found in the quarries • It can be found with welders • It can be found in electric stove and cookers • It can be found in fuel • It can be found in cooking pots • It can be found in chemical products • It can be found in local herbs
What risks do you think is associated with exposure to lead?	• It is poisonous • It can cause cancer	• It is dangerous to health • It can shorten life span • It can cause asthma • It can cause cough • It affects the eyes • It affects the chest
What do you think of when you hear the word lead poisoning?	• Death • Harm • Danger to health	• Disability • Lumps, burns and cancer • Blindness • Apollo • Retards physical growth • Reduces children's intelligence
Where do you think we can be exposed to lead?	• Fuel • Pipe borne water • Pencil •	• Soil • Plates • Tinned food • Bathing soap • Exhaust from industries
What other words have similar meaning to lead?	• Metals	•
What benefits can we derive from lead?	• Used to manufacture car battery	• To make electrical accessories • Tinkering
Are there any reasons why we might not be concerned about lead in our environment?	• Ignorance	•
Where can we get information about lead?	• Battery manufacturers • Welders • Battery Repairers	• Internet • Villages • Libraries

**Table 3:** Participants' attitudes and practices about lead exposure in Ibadan, Nigeria 2004

Have you ever tested the lead level in your domestic environment? If yes why did you do the test?	• No	
What are the reasons you might do a lead level test?	• If is going to be beneficial • To be educated about lead • I do not want to be at risk	• For financial benefit
What are the reasons you might not allow a lead level test to be carried out in your environment?	• Because lead is dangerous • Because I think it will be harmful	• For security reasons
What impact do you think lead or lead poisoning have on health?	• We do not know • It causes abdominal pain	• It makes people pale • It shortens life • It affects any body organ • It affects breathing • It affects intelligence • It affects productivity
How can we reduce the risks of lead exposure?	• NAFDAC (monitoring body for food and drug production in Nigeria) should monitor lead in the production processes • Nothing can be done about it	• By enlightenment through research and information • Through public awareness • Throw away all used batteries • Painters should cover their nostrils when painting • Government should sponsor and encourage lead research • Government should ban importation on used vehicles
